# US States with poor social health outcomes and more firearms have more morbidity due to mass shootings

**Published:** 2024-07

**Authors:** Lum Tony, Alex Huang, Megan E. Paul, Brian A. Coakley

**Affiliations:** ^*a*^ The Icahn School of Medicine at Mount Sinai. New York, NY. USA.

**Keywords:** Mass shootings, Social determinants of health, Firearm regulations morbidity, Death

## Abstract

**Background:**

Mass shootings represent a persistent public health crisis. Prior studies have linked social determinants of health (SDOH) to the phenomenon of gun violence, but there remain limited analyses on mass shooting events specifically.

**Methods:**

Mass shooting events from 2014-2019 were recorded from the Gun Violence Archive. State-level data regarding population, ATF registered weapons, federal firearm licensees and several SDOHs (poverty, unemployment and educational attainment) were collected from publicly-available US governmental databases. Giffords Law Center rankings were used to assess the relative strictness of each state’s gun laws. Gun ownership rates were obtained from the RAND Corporation. Bivariate analyses compared each SDOH, as well as ATF registered weapons, Giffords Center ranking and gun ownership rates, to the death rate, injury rate, and combined injury/death rate from mass shootings in each state. All associations were evaluated via Pearson’s Rho. Slope and p-values were analyzed, with a threshold significance value of p less than 0.05.

**Results:**

Unadjusted analysis revealed poor mental health, decreased educational attainment and increased unemployment to all be associated with an increased risk of mass shooting-related injury or death. Adjusted analysis revealed fewer firearm regulations, higher gun ownership, lack of handgun magazine restrictions and lack of long-gun registration requirements were associated with an increased risk of mass-shooting death. Similarly, adjusted analysis revealed lack of handgun permit requirements to be associated with both an increased risk of mass shooting-related injury and combined risk of injury/death.

**Conclusions:**

This study revealed associations between multiple SDOH and firearm restrictions with morbidity due to mass shooting events.

## Introduction

Mass shootings remain a persistent crisis in the United States.^1^ Each year, these events, which the U.S. Congress defines as firearm-related incidents in which 3 or more people are injured or killed, claim the lives of more than 300 people and injure over 1400.^2^ In addition, these shootings can have lasting effects on the psychological health of surviving victims and inflict a tremendous economic toll on affected communities.^3^ Although the financial impact specific to mass shootings has not been calculated, the cost of gun violence altogether amounts to over $229 billion US annually.^4^

Previous studies have found associations between measures of gun ownership, firearm-control laws and multiple social determinants of health (SDOH) with rates of gun violence.^5^ In addition, other studies have documented that efforts to reduce ease of firearm acquisition, including eliminating the need for identification and background checks, were associated with increased firearm mortality.^6^ Furthermore, a 2019 study revealed firearm-related homicides were associated with wealth inequality, level of citizens' trust in institutions, economic opportunity and public welfare spending.^5^

Although mass shooting events have been studied less intensively than gun violence overall, this gap has been diminishing. In particular, multiple studies have now shown an association between more firearm regulations and decreased morbidity due to mass shootings.^7-9^ However, other studies have painted more of a mixed picture. Specifically, while some analyses have demonstrated a correlation between firearm ownership and the incidence of mass shooting morbidity,^10-12^ others have failed to demonstrate any such similar links between restrictiveness of state-level firearm laws,^13^ federal assault weapons bans^14^ or right-to-carry laws^15^ on the incidence of mass shootings. Even more limited has been analyses analyzing the associations between SDOH and mass shooting events. Further, the paucity of studies that have been conducted have to date yielded conflicting results.^5,16^

As such, there exists a growing public health need to identify whether the same factors associated with overall gun violence are also correlated with mass shooting events specifically. Thus, we set out to determine if gun ownership rates, surrogate markers for the numbers of guns-in-circulation and multiple SDOH were associated with an increased risk of being injured or killed in a mass shooting event in the US.

## Methods 

**Outcomes:**

All mass shooting events from 2014-2021 were recorded from the online database of the Gun Violence Archive (GVA).^2^ The GVA is a non-profit organization which collects data from over 7,500 sources including media, law enforcement as well as both commercial and government organizations to compile a comprehensive database of US mass shooting events. This database has been utilized by a host of prior studies to quantify the toll of gun violence in states and communities.^17-20^ Injury and mortality data were also aggregated by state. State-level population data from the US Census Bureau was used to compute rates of injury or death per 100,000 persons.^21^

**Exposures:**

For each US state, gun ownership rates among adults were taken from the RAND corporation. The RAND corporation, a non-profit research organization, produces estimates of gun ownership at the state level through the use of composite survey data as well as four proxy indicators of gun ownership. These proxy indicators include the proportion of suicides in which a gun is used (from the Centers for Disease Control and Prevention [CDC]), the number of hunting licenses per capita (from the U.S. Fish and Wildlife Service), the number of Guns & Ammo magazine subscriptions per 100 residents (from the Alliance for Audited Media) and the number of background checks conducted per ten residents (from the National Instant Criminal Background Check System).^22^ The Bureau of Alcohol, Tobacco, Firearms and Explosives (ATF) tracks the number of ATF registered weapons and FFLs for each state.^23-24^ ATF registered weapons data includes all weapons covered by the National Firearms Act of 1934. Specifically, the Act covers shotguns and rifles having barrels less than 18 inches in length, machine guns, firearm mufflers and silencers, and alternative firearms described as “any other weapons.^25^ FFLs are businesses involved in dealing, manufacturing, and/or importing firearms or ammunition. These businesses include gunsmiths, pawnbrokers, dealers, manufacturers, importers and collectors. The Giffords Law Center to Prevent Gun Violence (GLCPGV) scorecard rankings were used to quantify the relative strictness of each state’s firearm laws.^26^ Specifically, the GLCPGV ranks each state from 1-50, with higher numbers indicating more lenient gun laws. Each state’s GLCPGV ranking was recorded for each year from 2014 to 2021 and subsequently averaged. For example, a GLCPGV scorecard ranking of 50 would indicate the most lenient firearm laws in the county, while a ranking of 1 would denote the most restrictive. Additionally, for each state, the average total number of firearm provisions and the presence or absence of handgun magazine size restrictions, assault weapons bans, long gun registration requirements, and handgun permit requirements from 2014-2021 were recorded.^27^ Multiple prior studies have validated the GLCPGV rankings as a measure of the relative restrictiveness of state-level firearm control laws.^28-33^

**Covariates**

State level data was also collected for multiple SDOH and potential surrogate markers of firearms-in-circulation. Data pertaining to poor mental health, race and educational attainment for each state was collected for the years 2014-2021. Mental health data was taken from the CDC’s Behavioral Risk Surveillance System.^34^ Specifically, poor mental health was defined as the percentage of adults having ≥ 14 days of poor mental health within the prior month, as has been done in other studies.^35^ Racial data was collected from the US Census Bureau.^36^ Educational attainment data, specifically the accrual of a bachelor’s degree or higher, was taken from the National Center for Education Statistics.^37^

**Statistical Analysis:**

First, descriptive statistics were calculated for each of the firearm-related measures and compared across states. Unadjusted analyses were conducted comparing the aforementioned SDOH, surrogate markers for firearms-in-circulation and relative strictness of existing gun laws to mass shootings outcomes. These analyses were repeated using mass shooting-related injuries, deaths and combined rates of injury/death as the outcomes. Next, multivariable (adjusted) linear regression analyses were used to compare each of the SDOH, firearm restrictiveness measures, and measures of firearms-in-circulation with (1) deaths, (2) injuries, and (3) combined injury/death by state as the outcomes. During this adjusted analyses, we specifically controlled for race, poor mental health, educational attainment, unemployment and poverty.

All associations were evaluated via Pearson’s Rho. The slope (β), 95% confidence intervals (CI) and p values were analyzed for each comparison, with a threshold significance value of p<0.05. All analyses were conducted in R Studio version 1.4.1717. 

## Results

Based on GLCPGV rankings, California was determined to have the most stringent gun laws, whereas Mississippi had the most lenient.^26^ Vermont had the highest concentration of ATF registered weapons per capita (5174.04 per 10k residents) while Rhode Island had the lowest (41.29 per 10k residents). Montana also had the highest rate of FFL’s (15.63 per 10k residents) whereas New Jersey had the lowest (0.67 per 10k residents). Finally, gun ownership was highest in Alaska (61.7%) and lowest in Delaware (5.2%). 

Hawaii and North Dakota had no mass shooting events over the time period studied. Additionally, Rhode Island and New Hampshire had no mass shooting-related deaths. Among those with at least one mass shooting-related death, Massachusetts had the lowest average rate of individuals killed (0.03 per 100k residents) whereas Mississippi had the highest rate of individuals killed (0.38 per 100k residents). Maine had the lowest rate of mass shooting-related injuries (0.02 per 100k residents) while Nevada had the highest rate of mass shooting-related injuries (2.23 per 100k residents). When combining mass shooting-related injuries and deaths, New Hampshire had the lowest average rate of injury/death (0.04 per 100k residents) while Nevada had the highest (2.58 per 100k residents), Table 1 . 

**Table 1:** Firearm-Related Statistics by State

States	Population	Killed per 100k	Injured per 100k	K+I per 100k	Mass Shooting Events per 100k	ATF registered weapons per 10k	ATF federal firearm licensees per 10k	% of adults owning firearms	Giffords Gun Law Ranking
Alabama	4,913,028	0.24	0.85	1.10	0.21	308.31	4.34	48.9	37
Alaska	736,091	0.10	0.31	0.41	0.07	242.22	14.85	61.7	42
Arizona	7,053,341	0.11	0.17	0.28	0.05	125.43	3.64	32.3	45
Arkansas	3,000,820	0.14	0.71	0.86	0.16	607.24	5.99	57.9	40
California	39,207,386	0.12	0.40	0.52	0.10	89.34	1.90	20.1	1
Colorado	5,624,751	0.15	0.40	0.54	0.09	176.29	4.36	34.3	15
Connecticut	3,586,326	0.05	0.40	0.45	0.08	184.70	4.38	16.6	3
Delaware	964,455	0.17	0.76	0.93	0.18	560.13	2.89	5.2	11
Florida	20,971,471	0.17	0.54	0.71	0.11	172.87	2.94	32.5	24
Georgia	10,450,545	0.17	0.59	0.76	0.15	197.86	3.16	31.6	32
Hawaii	1,427,901	0.00	0.00	0.00	0.00	56.99	1.74	14.2	4
Idaho	1,746,673	0.03	0.04	0.07	0.01	192.97	7.44	56.9	48
Illinois	12,776,338	0.25	1.50	1.75	0.34	42.39	3.54	26.2	8
Indiana	6,690,562	0.15	0.49	0.64	0.12	207.78	4.15	33.8	27
Iowa	3,149,397	0.04	0.26	0.31	0.06	382.90	5.90	33.8	20
Kansas	2,916,195	0.14	0.35	0.49	0.08	171.18	5.98	32.2	43
Kentucky	4,459,204	0.10	0.44	0.54	0.10	186.06	5.06	42.4	46
Louisiana	4,655,572	0.35	1.73	2.08	0.37	233.42	4.09	44.5	36
Maine	1,343,596	0.11	0.02	0.13	0.02	280.71	8.53	22.6	33
Maryland	6,049,917	0.18	0.81	0.99	0.19	184.03	4.09	20.7	6
Massachusetts	6,875,097	0.03	0.24	0.27	0.05	23.17	4.25	22.6	7
Michigan	9,982,907	0.14	0.50	0.63	0.12	70.97	4.29	28.8	21
Minnesota	5,586,161	0.06	0.40	0.46	0.08	151.35	4.71	36.7	14
Mississippi	2,977,535	0.38	1.02	1.40	0.24	262.40	5.07	42.8	50
Missouri	6,113,076	0.25	0.86	1.11	0.21	71.32	7.58	27.1	47
Montana	1,058,554	0.16	0.04	0.20	0.05	201.03	15.63	52.3	35
Nebraska	1,922,297	0.06	0.31	0.37	0.07	832.49	5.77	19.8	19
Nevada	2,992,698	0.35	2.23	2.58	0.11	53.55	3.81	37.5	17
New Hampshire	1,355,007	0.00	0.04	0.04	0.01	186.54	7.34	14.4	30
New Jersey	8,974,586	0.08	0.49	0.57	0.12	54.45	0.67	11.3	2
New Mexico	2,098,364	0.23	0.33	0.56	0.10	337.99	5.03	49.9	18
New York	19,684,802	0.05	0.44	0.49	0.10	51.41	2.01	10.3	5
North Carolina	10,284,073	0.14	0.41	0.55	0.10	81.58	3.87	28.7	25
North Dakota	759,805	0.00	0.00	0.00	0.00	1022.73	8.65	47.9	38
Ohio	11,680,193	0.16	0.63	0.78	0.14	142.25	3.88	19.6	26
Oklahoma	3,937,335	0.07	0.26	0.33	0.07	188.24	5.67	31.2	39
Oregon	4,139,169	0.07	0.21	0.28	0.04	159.62	5.79	26.6	16
Pennsylvania	12,844,058	0.13	0.62	0.75	0.14	201.93	4.44	27.1	13
Rhode Island	1,066,928	0.00	0.26	0.26	0.05	41.29	4.22	5.8	9
South Carolina	5,031,597	0.26	0.81	1.07	0.19	180.33	3.58	44.4	31
South Dakota	873,288	0.21	0.09	0.29	0.06	304.62	8.29	35	44
Tennessee	6,747,747	0.17	0.85	1.02	0.19	158.03	4.46	39.4	29
Texas	28,382,495	0.17	0.40	0.57	0.09	220.09	3.28	35.7	34
Utah	3,130,504	0.05	0.07	0.12	0.02	253.66	3.60	31.9	28
Vermont	629,580	0.08	0.00	0.08	0.02	5174.04	9.01	28.8	23
Virginia	8,484,641	0.12	0.45	0.57	0.11	7.51	4.40	29.3	12
Washington	7,442,497	0.10	0.18	0.28	0.05	137.70	3.36	27.7	10
West Virginia	1,814,119	0.09	0.08	0.18	0.03	375.22	7.95	54.2	41
Wisconsin	5,810,186	0.10	0.30	0.39	0.07	60.85	4.87	34.7	22
Wyoming	580,592	0.04	0.04	0.09	0.02	2240.27	14.26	53.8	49

In the unadjusted analyses comparing each of the SDOH, firearm provisions and measures of gun ownership, higher percentages of non-White citizens (β=0.17, p value=0.03), poorer mental health (β=1.88, p value=0.008), lower educational attainment (β=-0.007, p value=0.002), increased unemployment (β=4.01, p value=0.002) higher GLCPGV ranking (β=0.002, p value=0.04) and no long gun registration requirements (β= -0.11, p value=0.03) were associated with higher rates of mass shooting-related deaths. In the adjusted analyses controlling for race, poor mental health, educational attainment, unemployment, and poverty, fewer total firearm provisions (β=-0.001, p value=0.04), higher gun ownership (β=0.27 p value=0.04), no handgun magazine size restrictions (β=-0.14 p value=0.03) and no long gun registration requirements (β=-0.11, p value=0.03) were statistically associated with higher rates of mass shooting-related deaths, Table 2 . To interpret these results, for example, each 1 unit increase in gun ownership is associated with a 0.27-unit increase in the risk of mass-shooting death after controlling for race, poor mental health, educational attainment, unemployment and poverty. 

**Table 2:** Firearm-Related Variables and SDOH vs. Rate of Mass-Shooting Related Deaths

Exposure	Unadjusted		Adjusted	
	Beta	P Value	Beta	P Value
Gun Ownership	0.15	0.12	0.27	0.04
ATF-Registered Weapons	-1.69E-06	0.68	-2.26E-06	0.54
ATF FFL’s	-0.0005	0.25	3.01E-06	0.99
Firearm Provisions	-0.001	0.04	-0.001	0.04
Assault Weapons Bans	-0.05	0.2	-0.03	0.5
Long Gun Registration Requirements	-0.11	0.03	-0.11	0.03
Handgun Permit Required	0.001	0.98	-0.01	0.56
Handgun Magazine Size Restrictions	-0.12	0.07	-0.14	0.03
GLCPGV	0.002	0.04	0.002	0.15
Race	0.17	0.03	–	–
Poor Mental Health	1.88	0.008	–	–
Educational Attainment	-0.007	0.002	–	–
Unemployment	4.01	0.002	–	–
Poverty	-0.002	0.99	–	–

– Indicates that the metric was controlled for in the adjusted model GLCPGV = Giffords Law Center to Prevent Gun Violence state rankings

Unadjusted analyses revealed that more FFL’s (β=0.006, p value=0.01), higher percentages of non-White citizens (β=0.95, p value=0.02), poorer mental health (β=7.86, p value=0.02), increased unemployment (β=23.74, p value=<0.001) and no handgun permit requirements (β=-0.21, p value=0.04) were associated with higher rates of mass shooting-related injuries. Adjusted analyses revealed that states with no handgun permit requirement (β=-0.27, p value=0.01) were statistically associated with higher rates of mass shooting-related injuries Table 3.

**Table 3:** Firearm-Related Variables and SDOH vs. Rate of Mass-Shooting Related Injuries

Exposure	Unadjusted		Adjusted	
	Beta	P Value	Beta	P Value
Gun Ownership	-0.27	0.57	-0.4	0.57
ATF-Registered Weapons	-1.73E-05	0.39	-1.41E-05	0.46
ATF FFL’s	0.006	0.01	-0.003	0.19
Firearm Provisions	0.0004	0.88	0.0009	0.76
Assault Weapons Bans	-0.003	0.99	0.03	0.9
Long Gun Registration Requirements	-0.27	0.32	-0.34	0.19
Handgun Permit Required	-0.21	0.04	-0.27	0.01
Handgun Magazine Size Restrictions	-0.37	0.28	-0.54	0.13
GLCPGV	-0.002	0.72	-0.006	0.28
Race	0.95	0.02	–	–
Poor Mental Health	7.86	0.02	–	–
Educational Attainment	-0.02	0.06	–	–
Unemployment	23.74	<0.001	–	–
Poverty	-0.62	-0.79	–	–

– Indicates that the metric was controlled for in the adjusted model GLCPGV = Giffords Law Center to Prevent Gun Violence state rankings

Unadjusted analyses revealed that more FFLs (β=0.006, p value=0.02), higher rates of non-White citizens (β=1.12, p value=0.01), poorer mental health (β=9.74, p value=0.02), lower levels of educational attainment (β=-0.03, p value=0.03), and increased unemployment (β=27.75, p value=<0.001) were associated with an increased combined rates of injury/death due to mass shootings. Adjusted analyses revealed that absence of a handgun permit requirement (β=-0.29, p value=0.03) was statistically associated with higher rates of combined injury/death due to mass shootings, Table 4.

**Table 4:** Firearm-Related Variables and SDOH vs. Rate of Mass-Shooting Related Injuries and Deaths Combined.

Exposure	Unadjusted		Adjusted	
	Beta	P Value	Beta	P Value
Gun Ownership	-0.12	0.83	0.87	-0.13
ATF-Registered Weapons	-1.90E-05	0.42	-1.63E-05	0.45
ATF FFL’s	0.006	0.02	-0.003	0.26
Firearm Provisions	-0.0005	0.83	-0.0002	0.95
Assault Weapons Bans	-0.05	0.81	-0.002	0.99
Long Gun Registration Requirements	-0.38	0.22	-0.45	0.14
Handgun Permit Required	-0.21	0.16	-0.29	0.03
Handgun Magazine Size Restrictions	-0.49	0.22	-0.68	0.1
GLCPGV	2.00E-04	0.96	-0.005	0.49
Race	1.12	0.01	–	–
Poor Mental Health	9.74	0.02	–	–
Educational Attainment	-0.03	0.03	–	–
Unemployment	27.75	<0.001	–	–
Poverty	-0.63	0.82	–	–

– Indicates that the metric was controlled for in the adjusted model GLCPGV = Giffords Law Center to Prevent Gun Violence state rankings

## Discussion

Our analysis revealed that states with poor mental health, lower educational attainment, higher unemployment, increased rates of gun ownership, and more lenient gun-control laws had higher rates of death and injury due to mass shootings. To our knowledge, this is one of the first studies demonstrating the association between mass shootings and multiple SDOH. Interestingly, our study did not find an association between assault weapons bans and reduced mass shooting casualties, despite the fact that assault weapons are often employed to carry out such acts. This may point to the difficulty with trying to isolate factors associated with an event that affects only a few thousand persons each year. In addition, given that it is often easy for people to transport firearms across state lines, this could certainly help explain why an assault weapons ban enacted by one state might not clearly be associated with a reduction in mass shooting mortality in that same state. Further, our study’s adjusted analysis revealed handgun magazine restrictions and long gun registration requirements to be associated with fewer mass shooting deaths, while handgun permit requirements were associated with reduced rates of mass shooting injury and combined rates of injury/death. These results argue that a stronger set of firearm laws at the state level does indeed correlate with fewer mass shooting casualties. 

Prior studies have shown that lower socioeconomic status has been tied to increased gun violence, particularly in the adolescent population.^38^ Due to the design of our analysis, causal inferences cannot be made. However, we suspect that a negative feedback loop could be present. For instance, decreased educational attainment can lead to fewer employment opportunities and thus, poorer economic conditions. Those conditions can put an individual at higher risk for becoming the victim of a mass shooting event, which can leave them with increased psychological distress, reduced sense of safety and heightened financial burdens. This, in turn, can further worsen the socioeconomic status of said individuals. Similar to living victims of terrorist acts, surviving victims of mass shootings have been demonstrated to suffer from prolonged effects on both their physical and mental health.^39-40^

In summary, it seems prudent to suspect that the risk of a mass shooting event occurring in a specific state is undoubtedly multi-factorial. Some states have a more pervasive gun culture, which leads to more lenient firearm laws and increased firearm ownership. Perhaps unsurprisingly, some of these states also have an increased incidence of mass shooting events. On the other other hand, other states have enacted policies that have reduced unemployment rates and increased educational attainment among their populations, which may have had the indirect effect of reducing mass shootings for their communities. Given the interplay between these factors, it can be difficult to point to individual factors as being causative of or protective against such tragic occurrences. 

Our study does have several limitations. First, we were limited in the availability of data to represent each domain of SDOH. In addition, given that this is an ecological study, our findings can only argue association rather than causation. Further, not all firearms are subject to the National Firearms Act of 1934 and thus, tracked by the ATF. For example, pistols or revolvers having rifled bores are not captured in the ATF registered weapons statistics. Thus, the number of ATF registered weapons is not a fully comprehensive measure of the number of publicly-owned guns. Further, analyzing metrics at the state level means that would not be able to conduct a more granular analysis as to how certain measures, such as poverty or firearm prevalence, might vary significantly within different parts of the same state. To that end, Reeping et al. recently conducted an analysis showing that the risk of firearm-related death is disproportionately worse in rural US counties, when compared to urban county counterparts.^41^ However, many of the measures evaluated in our study are only reliably reported at the state level, making a more granular analysis unfeasible. Finally, given the retrospective nature of our analysis, our findings are potentially susceptible to unmeasured confounding. 

## Conclusion

This analysis demonstrates an association between several state-level measures of SDOH and morbidity from mass shooting events. This study represents one of the first to shed light on such a link. Future research should take a more granular approach to determine which individual policies regarding SDOH and firearm regulation would be the most efficacious in reducing the impact of mass shootings in the US.

**Acknowledgment:**

The authors would like to acknowledge all of the lives that have been lost due to mass shooting events in US and globally.

**Author contributions:**

TL and AH contributed to data accrual, data analysis and writing the manuscript. MP helped design the study, conducted data analysis and interpretation of the findings. BC helped design the study, accrue study data, interpret the statistical analysis and writing of the manuscript. All authors contributed to editing and approving the final manuscript.

## References

[1] Peña PA, Jena A. Mass Shootings in the US During the COVID-19 Pandemic. JAMA Netw Open. 2021 Sep; 4(9):e2125388-e2125388. 10.1001/jamanetworkopen.2021.25388 PMC8446816 34529068

[2] Gun Violence Archive. https://www.gunviolencearchive.org/, accessed 4 July 2023.

[3] Shultz JM, Thoresen S, Flynn BW, Muschert G, Shaw J, Espinel Z, Walter F (2014). Multiple vantage points on the mental health effects of mass shootings. Curr Psychiatry Rep.

[4] U.S. Congress Joint Economic Committee Democratic Staff. A State-by-State Examination of the Economic Costs of Gun Violence 2019. https://www.jec.senate.gov/public/_cache/files/9872b4d4-4151-4d3e-8df9-bc565743d990/economic-costs-of-gun-violence---jec-report.pdf, accessed 1 December 2020.

[5] Kim D. Social determinants of health in relation to firearm-related homicides in the United States: A nationwide multilevel cross-sectional study. PLoS Med. 2019 Dec 17;16(12):e1002978. 10.1371/journal.pmed.1002978 PMC6917210 31846474

[6] Kalesan B, Mobily ME, Keiser O, Fagan JA, Galea S (2016). Firearm legislation and firearm mortality in the USA: a cross-sectional, state-level study. Lancet.

[7] Webster DW, McCourt AD, Crifasi CK, Booty MD, Stuart EA (2020). Evidence concerning the regulation of firearms design, sale, and carrying on fatal mass shootings in the United States. Criminol Public Policy.

[8] Siegel M, Goder-Reiser M, Duwe G, Rocque M, Fox JA, Friedel EE (2020). The relation between state gun laws and the incidence and severity of mass shootings in the United States, 1976 to 2018. Law Hum Behav.

[9] Tiderman L, Dongmo NF, Munteanu K, Kirschenbaum M, Kerns L (2023). Analyzing the impact of state gun laws on mass shootings in the United States from 2013 to 20201. Public Health.

[10] Daraklis M, Pol M, Johnson L, Salvatora C, Kerns L (2024). A Statistical Analysis of the Impact of Gun Ownership on Mass Shootings in the USA Between 2013 and 2022. J Urban Health.

[11] Reeping PM, Cerda M, Kalesan B, Wiebe DJ, Galea S, Branas CC (2019). State gun laws, gun ownership, and mass shootings in the US: cross-sectional time series. BMJ.

[12] Lemieux F (2014). Effect of Gun Culture and Firearm Laws on Gun Violence and Mass Shootings in the United States: A Multi-Level Quantitative Analysis. International Journal of Criminal Justice Sciences.

[13] Duchesne J, Taghavi S, Torah E, Simpson JT, Tatum D (2022). State Gun Law Grades and Impact on Mass Shooting Event Incidence: An 8-Year Analysis. J Am Coll Surg.

[14] DiMaggio C, Avraham J, Berry C, Bukur M, Feldman J, Klein M (2019). Changes in US mass shooting deaths associated with the 1994-2004 federal assault weapons ban: Analysis of open-source data. J Trauma Acute Care Surg.

[15] Duwe G, Kovandzic T, Moody CE (2002). The Impact of Right-to-Carry Concealed Firearm Laws on Mass Public Shootings. Homicide Studies.

[16] Cabrera JF, Kwon R (2018). Income Inequality, Household Income, and Mass Shooting in the United States. Front Public Health.

[17] Shipley J, Grigorian A, Swentek L, Barrios a, Kuza C, Santos J (2024). Long gun violence in California versus Texas: How legislation can reduce firearm violence. Surg Open Sci.

[18] Beaulieu-Jones B, Sunkara N, Kenzik K, Davis ES, Torres CM, Seamon MJ (2024). Nearly 20 Years Since the Federal Ban: Can State-Level Assault Weapon Prohibitions Fill the Void? Comparative Analysis of Case Fatality and Assault Weapon Recovery in States with and Without an Assault Weapon Ban. J Surg Res.

[19] Ward JA, Cepeda JA, Jackson DB, Crifasi CK (2024). Characteristics of Injuries Shootings by Police Along the Urban-Rural Continuum. Am J Prev Med.

[20] Bruns A, Aubel A, Zhang X, Buggs S, Kravitz-Wirtz N (2024). Community exposure to gun homicide and adolescents’ educational aspirations. J Adolesc.

[21] Bureau UC. Population and Housing Unit Estimates Datasets.; 2023. https://www.census.gov/programs-surveys/popest/data/data-sets.html, accessed 4 July 2023.

[22] Schell TL, Peterson S, Vegetabile BG, Scherling A, Smart R, Morral AR. State-Level Estimates of Household Firearm Ownership. RAND Corporation; 2020.

[23] Data & Statistics | Bureau of Alcohol, Tobacco, Firearms and Explosives. Published 2023. https://www.atf.gov/resource-center/data-statistics, accessed 4 July 2023.

[24] Federal Firearms Listings | Bureau of Alcohol, Tobacco, Firearms and Explosives. https://www.atf.gov/firearms/listing-federal-firearms-licensees, accessed 1 September 2023.

[25] U.S. Code § 5849 - Citation of chapter | U.S. Code | US Law | LII / Legal Information Institute. https://www.law.cornell.edu/uscode/text/26/5849, accessed 4 July 2023.

[26] Gun Laws | Giffords. https://giffords.org/lawcenter/gun-laws/, accessed 4 July 2023.

[27] McClenathan J, Pahn M, Siegel M. STATE FIREARM LAWS.; 1991. http://statefirearmlaws.orghttp://statefirearmlaws.orgiihttp://statefirearmlaws.org, accessed 4 July 2023.

[28] Reeping PM, Morrison CN, Rudolph KE, Goyal MK, Branas CC (2021). A comparison and analysis of seven gun law permissiveness scales. Inj Epidemiol.

[29] Paul M, Tomlinson A, Zhang R, Liu B, Coakley BA. Firearm-Related Juvenile Death Rates Correlate With Gun Ownership Rates, Measures of Guns in Circulation, and Leniency of Existing Firearm Laws Among US States. J Surg Res. 2024 Aug;300:381-388. PMID: 388848639 10.1016/j.jss.2024.04.078 38848639

[30] Paul M, Kahan A, Coakley BA (2024). Modeling the Relationship Between Firearm Restrictions, Medicaid Expansion, and Political Partisanship with Pediatric Firearm Mortality Costs. J Pediatr Surg.

[31] Paul M, Coakley BA (2023). State Gun Regulations and Reduced Gun Ownership are Associated with Fewer Firearm-Related Suicides Among Both Juveniles and Adults in the USA. J Pediatr Surg.

[32] Paladugu A, Shipley J, Grigorian A, Qazi A, Kong A, Kuza C (2024). Trends in Legal Firearm Transactions: A Possible Protective Role of Gun Law Strength. Am Surg.

[33] Chammas M, Byerly S, Lynde J, Mantero A, Saberi R, Gilna G (2023). Association Between Child Access Prevention and Sate Firearm Laws with Pediatric Firearm-Related Deaths. J Surg Res.

[34] 2021 BRFSS Survey Data and Documentation. https://www.cdc.gov/brfss/annual_data/annual_2021.html, accessed 4 July 2023.

[35] Massetti GM, Thomas CC, King J, Ragan K, Buchanan Lunsford N (2017). Mental Health Problems and Cancer Risk Factors Among Young Adults. Am J Prev Med.

[36] U.S. Census Bureau QuickFacts: United States. https://www.census.gov/quickfacts/fact/table/US/PST045222, accessed 4 July 2023.

[37] NCES Fast Facts. National Center for Education Statistics. https://nces.ed.gov/fastfacts/display.asp?id=27, accessed 4 July 2023.

[38] Bushman BJ, Newman K, Calvert SL, Downey G, Dredze M, Gottfredson M (2016). Youth violence: What we know and what we need to know. Am Psychol.

[39] Richardson JD, Davidson D, Miller FB (1996). After the shooting stops: follow-up on victims of an assault rifle attack. J Trauma.

[40] Stene LE, Dyb G (2015). Health service utilization after terrorism: A longitudinal study of survivors of the 2011 Utøya attack in Norway. BMC Health Serv Res.

[41] Reeping P, Mark A, Branas C, Gobaud A, Nance M (2023). Firearm Death Rates in Rural vs Urban US Counties. JAMA Surg.

